# Comparing multilevel and Bayesian spatial random effects survival models to assess geographical inequalities in colorectal cancer survival: a case study

**DOI:** 10.1186/1476-072X-13-36

**Published:** 2014-10-04

**Authors:** Paramita Dasgupta, Susanna M Cramb, Joanne F Aitken, Gavin Turrell, Peter D Baade

**Affiliations:** Cancer Council Queensland, PO Box 201, Spring Hill, QLD 4004 Australia; School of Mathematical Sciences, Queensland University of Technology, 2 George St, Brisbane, QLD 4000 Australia; Griffith Health Institute, Griffith University, Gold Coast Campus, Parklands Drive, Southport, QLD 4222 Australia; School of Public Health and Social Work, Queensland University of Technology, Herston Road, Kelvin Grove, QLD 4059 Australia

**Keywords:** Bayesian, Multilevel, Colorectal cancer, Epidemiology, All-cause survival, Spatial

## Abstract

**Background:**

Multilevel and spatial models are being increasingly used to obtain substantive information on area-level inequalities in cancer survival. Multilevel models assume independent geographical areas, whereas spatial models explicitly incorporate geographical correlation, often via a conditional autoregressive prior. However the relative merits of these methods for large population-based studies have not been explored. Using a case-study approach, we report on the implications of using multilevel and spatial survival models to study geographical inequalities in all-cause survival.

**Methods:**

Multilevel discrete-time and Bayesian spatial survival models were used to study geographical inequalities in all-cause survival for a population-based colorectal cancer cohort of 22,727 cases aged 20–84 years diagnosed during 1997–2007 from Queensland, Australia.

**Results:**

Both approaches were viable on this large dataset, and produced similar estimates of the fixed effects. After adding area-level covariates, the between-area variability in survival using multilevel discrete-time models was no longer significant. Spatial inequalities in survival were also markedly reduced after adjusting for aggregated area-level covariates. Only the multilevel approach however, provided an estimation of the contribution of geographical variation to the total variation in survival between individual patients.

**Conclusions:**

With little difference observed between the two approaches in the estimation of fixed effects, multilevel models should be favored if there is a clear hierarchical data structure and measuring the independent impact of individual- and area-level effects on survival differences is of primary interest. Bayesian spatial analyses may be preferred if spatial correlation between areas is important and if the priority is to assess small-area variations in survival and map spatial patterns. Both approaches can be readily fitted to geographically enabled survival data from international settings.

**Electronic supplementary material:**

The online version of this article (doi:10.1186/1476-072X-13-36) contains supplementary material, which is available to authorized users.

## Background

The importance of understanding social inequalities in cancer survival is well recognized [1, 2], including the impacts of both residential area characteristics and individual-level risk factors [3–6]. Much of the interest in the impact of area-level effects on cancer outcomes has been driven by the emergence of statistical methods that are designed to model geographically-structured data, including multilevel discrete-time [7, 8] and more recently, Bayesian spatial [4, 5] survival models. Since practical usage of these terms can differ, in our context we define “multilevel” structure as having a clear hierarchical one-to-many relationship between area and individual-level variables [9].

Multilevel discrete-time survival models [7, 8] are designed to account for the nested structure of individuals within geographical areas. They allow the simultaneous estimation of individual and area-level effects by modelling complex sources of variation at different hierarchical levels [9, 10]. In this multilevel framework, observations from one geographical area are assumed to be statistically independent of those in another area, so any spatial associations between geographical areas are ignored [11].

In contrast, Bayesian spatial methods were developed to explicitly incorporate spatial associations between geographical areas while describing the geographical patterns across areas. Survival in this context can be modelled either at the individual-level [12, 13] or by aggregating the unit-record data across area and covariates of interest [4, 5]. Area-level spatial effects are captured through modelled random terms for which an uncertainty distribution (the “prior” distribution) are specified [14]. This approach incorporates information from adjacent regions to help overcome data sparseness and account for between-area spatial associations. To date the widespread application of spatial models using large unit record datasets has been limited, since they usually require more advanced programming skills than typically required for standard statistical software packages and are computationally demanding [12, 13]. With a view to considering applications to large population-based cancer registry data, we chose instead to estimate the spatial survival inequalities by fitting generalised linear spatial models [15] to aggregated data. These models can be readily implemented with freely available software packages [4, 5].

Since the multilevel discrete-time and aggregated Bayesian spatial survival approaches require the researcher to ignore either the spatial or multilevel effects, respectively, the possibility that both effects may be simultaneously present in geographically structured data is overlooked. Hence multilevel models have been criticized in some instances for their inability to account for spatial dependencies of health outcomes [11, 16, 17]. The potential implications of adjusting solely for either multilevel or spatial effects in the context of geographical inequalities in health have however not been widely explored.

The literature on incorporating a spatial perspective into the multilevel setting is sparse and limited to small-scale studies [18–21]. For example multilevel membership models use additional random terms to model spatial clustering of neighboring areas with separate random terms used for each neighboring unit for each observation from a specific region [19, 22]. However the sheer number of random terms makes it computationally intractable to estimate these models when there are large cohorts covering a multitude of geographic areas. Alternatively the multilevel framework has been combined with regression approaches specifically designed to account for the spatial dependency of the area-level residuals [20, 21]. These also have limitations, including their complexity, lack of statistical stability, computational demands and difficulties in the interpretation of resultant estimates, each of which pose conceptual and technical challenges to their widespread implementation. We were unable to find any literature on integrated multilevel-spatial survival models. Nor, to our knowledge, have there been any studies that have used a case-study approach to explore the implications of using multilevel discrete-time and Bayesian spatial survival models on the same cohort.

In our case study we apply the two analytical methods of multilevel discrete-time and Bayesian spatial survival models to a population-based cohort of colorectal cancer (CRC) patients and examine the relative merits of the two approaches. Our focus is not on statistically comparing the estimates obtained [23], but rather comparing the interpretation of the output generated by the two approaches, and to discuss the differing data transformations, model assumptions and parameterization required for both approaches, in addition to specific software and computing considerations. Given the increasing popularity of both methods to assess geographical disparities in health, and the increasing interest in using large, population-based administrative datasets to examine these disparities, this case study aimed to improve understanding of the relative merits of the two approaches.

## Methods

Approval for this study was obtained from the University of Queensland Social and Behavioral Sciences Ethical Review Committee and Queensland Health.

### Study cohort

Histologically verified cases of invasive CRC (ICD-O3: C18-C20, C21.8) among individuals aged 20–84 years diagnosed between January 1, 1997 and December 31, 2007 with complete address information who survived for at least one day after diagnosis (n = 22,727) was acquired from the state-wide population-based Queensland Cancer Registry [24]. Information extracted from pathology forms was used to obtain the American Joint Committee on Cancer categories [25] for the stage at diagnosis and surgical margins as described previously [3].

Residential address at diagnosis was geocoded and assigned to a Statistical Local Area (SLA) [3]. SLAs are administrative units that cover the whole state with no gaps or overlaps, and are typically responsible for local infrastructure and thus deemed to be socio-economically relevant to their residents. There were 478 SLAs in Queensland in 2006 with a median population of 5,810 (range 7: 77,523) and median area of 14 km^2^ (range 0.3:106,188). Geographic remoteness at CRC diagnosis was classified according to the 2006 Australian Standard Geographical Classification Remoteness Index [26] and area-level disadvantage measured by the Index of Relative Socioeconomic Advantage and Disadvantage [27].

### Survival data

Patients were followed for all-cause mortality status until 31^st^ December 2010 with annual matching to the Registrar of Births, Deaths and Marriages and the (Australian) National Death Index [24]. Survival was calculated in years from date of diagnosis to death or the study end point. Survival times were truncated at 5 years of follow up to allow efficient computation of the complex survival models and to be consistent with previous studies [3, 4].

### Statistical analysis

Multilevel analysis was carried out with MLwiN version 2.26 [28] (University of Bristol, UK) that requires a once-off purchase while spatial modelling was performed using the freely accessible WinBUGS version 1.4 [29]. Both packages carry out Markov chain Monte Carlo (MCMC) estimations, provide diagnostic tests and plots to visually assess convergence of resulting chains and allow specification of random effects, and can be interfaced with Stata (Statacorp LP, TX, USA), as well as R [30]. However WinBUGS allows greater flexibility in the number of chains and choice of priors specified by the user whereas MCMC models in MLwiN can only be fitted with default priors and run on a single chain.

#### Multilevel discrete-time survival

While continuous time approaches are most commonly used for survival analysis there are several advantages to discrete-time models, especially in multilevel settings with large public health data sets [3, 8]. Fitting multilevel survival models requires an initial data restructuring in which a record is created for each time point that an individual survives. Generating such an expanded person-time dataset using months or days rather than years would increase the original dataset by 12 to 300-fold. Given the size of our initial cohort and available computational resources it was not feasible to implement this additional expansion. Hence multilevel discrete-time survival models that employ years as the time variable considerably reduce both the size of the expanded dataset and the computational demands for the subsequent survival analysis. In addition, parameter estimates from multilevel discrete-time and continuous-time Cox survival models have been shown to be comparable in a number of studies [3, 8]. Thus discrete-time survival models are preferred in the multilevel framework [3, 8].

Multilevel discrete-time survival models were fitted to an expanded *person-period file* containing a sequence of binary responses for each individual from each year [8]. The data file specifically incorporates censoring into the analysis, in that a censored individual will have a sequence of zero’s for each year whereas one who dies has a value of one for the year of death and zero for previous years (Appendix 1). Multilevel discrete-time survival models estimate the unexplained variation within- and between-SLAs with the residuals for different areas assumed to be independent of each other. The hazard function from the multilevel discrete-time survival model describes the conditional probability of death in interval *t* given they were still alive in the previous interval [8]. When modeling the hazard with the logit link; the exponentiated coefficients are interpreted as odds ratios (OR).

#### Multilevel discrete-time survival model specification

The discrete-time hazard function (*h*_*tij*_) for follow-up interval *t* and individual *i* in the *j*^*th*^ SLA is defined as the probability of a death (*e*_*tij*_) occurring during the follow-up interval *t*, given that no death has occurred in a previous year, i.e.:


which is the standard response probability for a binary variable. Therefore multilevel discrete-time survival models are essentially logistic regression models with the response variable being the binary indicator *e*_*tij*_ in the *person-period file*. We fitted a multilevel random-effects logistic model which was specified as:


where *β*_*0*_ is an intercept for the *j*^*th*^ SLA that varies randomly across the SLAs, *x*_*tij*_ is a vector of covariates with coefficient *β* which represents the effect of covariates on the hazard at follow up interval *t* for individuals in the reference (baseline) category of each variable (the baseline hazard), *ml*_*u*_*j*_ is the random effect for each SLA *j* which is normally distributed with mean 0 and variance *ml*_*σ*^*2*^_*u*_ and f(t) is a function of follow-up interval used to model the baseline hazard on a logistic scale. A dummy variable was used for each time period: i.e. baseline logit hazard takes form *f*(*t*) = *f*1*D*1 + *f*2*D*2 + … + *f*5*D*5 [8]. Finally the model assumes constant hazards over each follow-up interval [8].

#### Bayesian spatial survival

We modified a previously described [4] Bayesian spatial Poisson model to analyse five year all-cause survival. This model is specified as:


where *d*_*mtj*_ is the observed number of deaths among the CRC cohort in the *m*^*th*^ stratum [across all included covariates], *t*^*th*^ follow-up interval and *j*^*th*^ SLA. The value *d*_*mtj*_ has a Poisson distribution with mean *μ*_*mtj*_, *y*_*mtj*_ is person-time at risk, *α*_*t*_ is a time-varying intercept, and *β*_*m*_ represents the coefficients of the vector of covariates *x*, *spat*_*u*_*j*_ is the unexplained spatial variation in the modeled count of deaths for each area *j* and *spat*_*v*_*j*_ is the unexplained non-spatial variation in the modeled deaths [31]. The total variation is:


where *spat_σ*^*2*^_*u(m)*_ is the marginal variance for the spatial effect and *spat_σ*^*2*^_*v*_ is the variance for the non-spatial effect.

The input data are aggregated by each combination of individual and area-level covariates at the SLA level. A Poisson distribution is assumed for the modeled outcome (the observed mortality count in each stratum), while aggregated survival time is included as an offset variable in the model. This is a piecewise exponential model, where the follow-up time is divided into distinct intervals and the hazard is assumed constant across each interval. If the time intervals are split at the occurrence of each event (death), the Poisson survival model is equivalent to the Cox proportional hazards model [32, 33]. We selected annual time intervals due to the size of the dataset. Similar to the multilevel discrete-time model, for each individual and time interval, death (the response) is defined as 1 if the individual dies within that interval and 0 otherwise. However unlike the multilevel discrete-time model deaths are then aggregated across each stratum prior to being modelled. This Bayesian spatial model includes separate terms for the spatially correlated (*spat*_*u*_*j*_) and the spatially uncorrelated unexplained variation (*spat*_*v*_*j*_), where *j* is the SLA. The spatial term depends upon geographical location and implies that neighboring areas influence each other more than non-neighbors [34] whereas the *spat_v*_*j*_ term accounts for variation which is independent of geographical location.

### Estimation of the survival models

#### Multilevel discrete-time

Models were estimated with MCMC simulations [22] in MLwiN 2.26 [28] (University of Bristol, UK) interfaced with Stata 12.0 (StataCorp, Texas) [35]. Default non-informative uniform priors (Appendix 1) were used for the fixed parameters and an inverse gamma distribution for the between-area variance. Parameter estimates were obtained from 80,000 iterations after discarding an initial 40,000 iterations. The underlying hazard was described by a dummy variable for each year [8]. A three step modeling strategy was adopted as described previously [3]. Truncating survival times to five years allowed efficient MCMC estimation of the multilevel discrete-time models after expansion for this relatively large dataset.

#### Bayesian spatial

Models were fitted with the MCMC algorithm within WinBUGS 1.4 software [29] interfaced with Stata 12.0 [36]. After a burn-in period of 250,000 iterations a further 100,000 iterations were monitored. Models were developed systematically: first we fitted a null model with only random effects, then we added all the individual covariates before including area disadvantage and remoteness, first separately and then together for the final fully adjusted model.

The spatial variance (*spat-u*_*j*_) was modeled with an intrinsic conditional autoregressive (CAR) prior [31] with the neighboring SLAs primarily defined based on common borders, as previously described [37]. Diffuse normal priors were chosen for the intercept and regression coefficients. Model specification was completed by assigning weakly informative hyperpriors to the two precision (inverse variance) parameters. Prior distributions and the associated sensitivity analyses are further described in Appendix 2. Model inferences were relatively insensitive to the choice of hyperpriors (Additional file 1).

### Model practicalities

Since single chain MCMC simulations were the only option within MLwiN [22], they were used for all analyses.

MCMC chain convergence (for both approaches) was assessed by visual inspection of the trace, density and autocorrelation plots of the posterior distributions for monitored parameters. Default diagnostic tests in MLwiN [22, 38] were used for multilevel discrete-time models and the Geweke test (p <0.01 criteria for non-convergence) for Bayesian spatial models [39]. Model residuals (both approaches) were also graphically examined for goodness-of fit.

Model fit within the set of multilevel discrete-time or Bayesian spatial models was evaluated using the Bayesian deviance information criterion (DIC) [40] with smaller DIC values (≤7) indicating improved fit. As DIC values are sensitive to the underlying data structure [41], these were not used for comparisons between approaches but rather for comparing models within each approach.

Parameter estimates from multilevel and spatial Poisson models are presented as odds ratios (OR) and relative risks (RR), respectively, with 95% credible intervals (CrI).

### Random effects

#### Multilevel discrete-time

The median odds ratio (MOR) [10] that expresses area-level variance from multilevel models on the odds ratio scale was used to quantify area-level survival variation (Appendix 1). The value of MOR is always ≥ 1 with larger values indicating greater geographical variation. The intraclass correlation coefficient (ICC) is often used to quantify the contribution of the area-level variance to the total variance in multilevel linear models. However, the use of such measures in the context of logistic regression is questionable and not recommended in standard multilevel literature due to problems in their computation and interpretation [10, 42–44]. Alternative measures include the median odds ratio (MOR) [10] that expresses area-level variance from multilevel models on the odds ratio scale. This was used to quantify area-level survival variation in the present study.

#### Bayesian spatial

The relative contribution of the spatial component to the total variance was calculated using the spatial fraction [5] (Appendix 2). If the spatial fraction is close to 1 the spatial effect dominates, otherwise if close to 0 the unstructured component dominates [5]. This measure allows quantification of the extent to which the unexplained variation is associated with geographical location.

### Differences between approaches

Table 1 summarizes the main features and differences between the multilevel discrete-time and Bayesian spatial approaches used in this case study. The assumptions, underlying concepts and interpretation of area-level effects for the two approaches are compared and contrasted in Table 2.

**Table 1:** **A comparison of multilevel discrete-time and Bayesian spatial survival models used in this case study**

	Multilevel	Bayesian spatial
Software	MLwiN 2.26^1^	WinBUGS version 1.4
Cost	Once-off purchase	Free
Available interfaces	Stata	Stata, SAS, R
Initial data structure		
*Retains multilevel structure (Patients nested in higher-level units)*	Yes	No
*Data expansion required*	Yes	No
Geographical Structure	None	Preserves adjacent areas
Explanatory variables	Unit Record Individual and higher-level	Aggregated at individual-level and higher-level
Modelled Outcome	Individual deaths	Aggregated deaths
Random Effects	Yes	Yes
Prior distributions	Gamma, Uniform	Any including Gamma, Uniform, CAR
Default Priors	Yes	requires user specification of priors; greater flexibility
Estimation Method: MCMC	Yes	Yes
Number of MCMC chains	Single only	Single (multiple allowed also)
Level of random effects	Individual and higher-level	Higher-level
Within-area correlation	Yes	No
Between-area correlation	No	Yes
Adjacency matrix	No	Yes
Computational efficiency ( 5 year data)^2^	5-7 days	5-7 days
Ease of Implementation	R equires prior data expansion	Requires specification of model including prior distributions
Diagnostic Tests/ convergence plots	Yes	Yes
Questions answered:		
*Do area- and individual-level factors impact survival for individual patients?*	Yes	No
*Extent to which between-individual variability is explained by covariates at both levels*	Yes	No
*Estimates unexplained area-level spatial variation after adjusting for parameters*	No	Yes
*Map spatial variation by small-areas*	No	Yes
Cross level interactions	Yes	No
Allow unit record individual-level inferences	Yes	No
Parameter estimates	Odds ratio (OR)	Relative risk (RR)

CAR: Conditional autoregressive prior; MCMC: Markov chain Monte Carlo.

1. Can also be run with MLwiN/WinBUGS interface.

2. On an Intel® Xeon® 2 Duo processor 64 bit CPU with 2.39 GHz processor speed and 24.0 GB RAM.

**Table 2:** **Assumptions, underlying concepts and interpretation of area-level effects: multilevel discrete-time and Bayesian spatial survival models**

	Multilevel discrete-time	Bayesian spatial
**Assumptions**		
Data structure	Data is hierarchically structured with individuals nested within geographical areas.	Data is assumed to be spatially structured at the aggregated level.
Individuals	Individuals (level 1) living in the same area (level 2) are assumed to be correlated	No individual-level data is retained
Hazard	Constant hazards over each follow-up interval.	Constant hazards over each follow-up interval.
Area-level effects	Area-level random effect is constant and normally distributed. Area-level random effects for different geographical areas are independent of each other; hence any spatial associations between neighboring areas are ignored.	Area-level random effect is not assumed to be constant; rather it depends on the spatial relationship between areas with the assumption that the mean outcome between two neighboring areas is more similar than that between two more distant areas.
Modelled outcome	These are essentially logistic regression models with the outcome variable being a binary indicator that gives the probability of a death occurring in a follow-up interval given that no death has occurred in the previous year.	A Poisson distribution is assumed for the modeled outcome (i.e. observed mortality count) in each aggregated stratum. However the usual assumption for a Poisson model, that the variance equals the mean, is relaxed since additional random effect parameters are included.
**Underlying concepts**		
Baseline hazard	The baseline hazard is modelled on the logistic scale as a function of the follow-up interval.	The baseline hazard is not specifically defined as this is a semi-parametric model.
Censoring	The censoring information is included. A censored individual has a sequence of zero’s for each year whereas a person who dies has a value of one for the year of death and zero for previous years.	The censoring information is included. A censored individual has a sequence of zero’s for each year whereas a person who dies has a value of one for the year of death and zero for previous years. However deaths are then aggregated acrosseach stratum.
Equivalence to Cox model	Multilevel logistic regression with expanded dataset is a good approximation to the Cox proportional hazard model [8].	The Poisson survival model is a good approximation to the Cox proportional hazards model [32, 33].
Spatial smoothing	No spatial smoothing is incorporated	Models borrow information from adjacent regions (termed ‘spatial smoothing’) to help overcome data sparseness, allow shrinkage towards overall risk, produce more robust estimates and account for between-area spatial associations [49].
Spatial structure	An individual’s probability of death is statistically dependent on their area of residence at diagnosis. Spatial proximity to other areas is not considered.	The spatial structure is encoded into the prior distribution specified for the random effects and requires the definition of relationships between spatially close SLAs [31]. The variable is assumed to be normally distributed relative to the neighbourhood mean.
Levels of variance	The total variance is partitioned at different levels: between individuals living in the same area (individual-level) and that between two different areas (area-level).	The overall variance cannot be decomposed over different analytical levels. However the 2 random effects at the area-level allow the variance to be partitioned into spatially structured and unstructured variance.
**Interpretation of the area-level random effects**		
Number	One type	Two types
Nature	Area-level random effects disregard any spatial correlation that may be present in the data and ignore the specific effect of location.	The spatially correlated area-level random effect assumes similarity between neighboring areas and quantifies the residual variation that is associated with geographical location. The uncorrelated or unstructured area-level random effect assumes independence between areas and allows for area-level variation that is not spatially correlated.

## Results

### Study population

The final cohort had a median age at diagnosis of 68 years and median follow-up time of 5.0 years with unadjusted 5-year all-cause survival of 58.1% (95% CI: 57–58) (Table 3). All covariates in Table 3 had significant bivariate associations with survival outcomes (log rank test: 0.001 ≤ p <0.003).

**Table 3:** **Cohort description and five year all-cause survival estimates for colorectal cancer patients, Queensland, 1997-2007**

Sub group	N (%)	% Deaths	All-cause survival [95% CI] ^1^	p
All patients in cohort	22,727	41.1	58.1 [57, 58]	
***Area-Remoteness Index of Australia (ARIA)***				*< 0.001*
Major city	13,155 (57.9)	39.6	59.6 [59, 60]	
Inner regional	5,139 (22.6)	41.4	57.8 [56, 59]	
Outer regional	3,485 (15.3)	45.1	54.1 [52, 56]	
Remote^2^	948 (4.2)	46.2	53.1 [50, 56]	
***Index of Relative socioeconomic advantage and disadvantage (IRSAD)***				*< 0.001*
Quintile 5 (least disadvantaged)	3,193 (14.1)	36.4	62.8 [61, 65]	
Quintile 4	5,101 (22.4)	38.9	60.2 [59, 62]	
Quintile 3	6,075 (26.7)	41.0	58.2 [57, 59]	
Quintile 2	5,335 (23.5)	44.5	54.6 [53, 56]	
Quintile 1 (most disadvantaged)	3,023 (13.3)	43.8	55.4 [54, 57]	
***Age group***				*< 0.001*
20 to 49	1,873 (8.2)	32.0	67.4 [65, 70]	
50 to 59	3,938 (17.3)	32.8	66.7 [65, 68]	
60 to 69	6,578 (28.9)	37.1	62.1 [61, 63]	
70-79	7,718 (34.1)	45.6	53.5 [52, 55]	
80-84	2,620 (11.5)	56.7	41.9 [40,44]	
***Gender***				*< 0.001*
Male	12,879 (56.7)	42.9	56.2 [55, 57]	
Female	9,848 (43.3)	38.8	60.6 [60, 62]	
***Indigenous status***				*< 0.001*
Non Indigenous	20,868 (91.8)	43.1	56.1 [55, 57]	
Indigenous	181 (0.8)	45.3	53.7 [45, 61]	
Not stated	1,678 (7.4)	16.7	82.9 [81, 85]	
***Marital status***				*<0.001*
Married	14,532 (63.9)	39.0	60.1 [59, 61]	
Never married/single	1,541 (6.8)	46.5	52.6 [50, 55]	
Widowed	3,951 (17.4)	48.2	51.1 [49, 52]	
Divorced	1,822 (8)	44.4	54.7 [52, 57]	
Separated	454 (2)	31.9	67.3 [63, 71]	
Not stated	427 (1.9)	20.6	79.3 [75, 83]	
***Occupation category***				*< 0.001*
Professional	4,783 (21.1)	48.6	50.6 [49, 52]	
White collar	2,665 (11.7)	52.6	46.7 [44,49]	
Blue collar	3,789 (16.7)	59.5	39.4 [38,41]	
Not in labor force	7,529 (33.1)	33.5	65.9 [65, 67]	
Not stated/Inadequately described	3,961 (17.4)	21.0	78.2 [77, 79]	
***Country of birth*** ^***3***^				*< 0.001*
Australia	17,367 (76.4)	41.9	57.2 [57, 58]	
Other English-speaking	4,580 (20.2)	39.2	60.2 [59, 62]	
Non-English-speaking	780 (3.4)	34.0	64.2 [61, 68]	
***Site*** ^***4***^				*=0.003*
Proximal (R) colon	7,874 (34.6)	41.8	57.5 [56, 59]	
Distal (L) colon	5,865 (25.9)	39.5	59.6 [58, 61]	
Colon NOS	1,299 (5.7)	54.0	45.3 [43,48]	
Rectal	7,689 (33.8)	39.4	59.8 [59, 60]	
***Stage***				*< 0.001*
Stage A	4,332 (19.1)	18.3	81.1 [80, 83]	
Stage B	6,323 (27.8)	28.9	70.3 [69, 71]	
Stage C	5,846 (25.7)	47.9	50.8 [50, 52]	
Stage D	2,576 (11.3)	84.7	13.9 [12,15]	
Unknown stage	3,650 (16.1)	47.4	51.9 [50, 54]	
***Differentiation***				*< 0.001*
Well differentiated	1,107 (4.9)	31.9	67.3 [65, 70]	
Moderately differentiated	13,953 (61.4)	36.7	62.4 [62, 63]	
Poorly differentiated	4,206 (18.5)	52.9	46.2 [45,48]	
Not stated	3,461 (15.2)	47.2	52.2 [50, 54]	
***Surgical margins***				*< 0.001*
Clear	16,664 (73.4)	36.3	62.9 [62, 64]	
Positive	530 (2.3)	39.8	59.7 [55, 61]	
Unknown	5,533 (24.3)	55.7	43.6 [43,45]	

CI = confidence interval; *p*-values calculated using log-rank test for equality of survivor functions restricting follow-up to five years for each patient.

1. From Kaplan-Meir survival analysis.

2. Includes remote and very remote categories.

3. Other English-speaking: those born in New Zealand, United Kingdom, Ireland, or North America; non-English-speaking: those not born in Australia, New Zealand, United Kingdom, Ireland or North America.

4. Colorectal sites defined as proximal colon (ICDO3: C180 to C184), distal colon (ICDO3: C185-C187), unspecified colon (ICDO3: C188-C189) and rectal (ICDO3: C19-C20, C218).

### Statistical analysis

The fully-adjusted main-effects multilevel discrete-time model (Model 5; Additional file 2) had the smallest DIC value indicating it had the best fit to the data and so was the preferred multilevel model. Similarly, the best-fitting model for the Bayesian spatial analysis (out of Models 7–13) was Model 11 (Additional file 3) which simultaneously adjusted for all aggregated individual- and area-level covariates and included both random effects. Based on the MCMC diagnostic tools, all monitored parameters converged for both multilevel discrete-time and Bayesian spatial models. No problems with model fit were detected on visual inspection of model residuals for both approaches.

Full adjustment for all considered covariates substantially reduced the residual geographical variation in survival for both approaches (Tables 4 and 5). The final multilevel discrete-time model had for example a non-significant area-level effect (p = 0.118) with an associated MOR of 1.07 (Table 4). For the spatial analysis the final smoothed RR estimates for all-cause deaths ranged from 0.86 to 1.20 (median 0.99) with CrIs that generally overlapped the average value of 1.00 (Additional file 4). This illustrates that much of the geographical variability in survival was accounted for by the included covariates. Only 55% of the variance in the fully adjusted Bayesian spatial model was spatially structured, and the estimated spatial fraction had a wide 95% CrI (35–73; Table 5). As the spatial fraction is the ratio of the marginal spatial structured variance to the sum of the variance of both marginal spatial structured and unstructured random effects, a value close to the midpoint of 0.5 suggests that neither the spatial or the unstructured effect is dominant.

**Table 4:** **Estimated area-level random effects from multilevel discrete-time survival models**

Model	Description ^1^	***ml***_***u***_***j***_^2^ (95% CrI)	***p*** ^3^	MOR (95% CrI) ^4^
1	*Null (no covariates) only individual & area-level random effects*	0.025 (0.014, 0.039)	<0.001	1.16 (1.13, 1.21)
2	*Individual-level covariates*	0.011 (0.006, 0.018)	0.04	1.10 (1.08, 1.14)
3	*Individual-level covariates & area-remoteness*	0.007 (0.003, 0.014)	0.10	1.08 (1.05, 1.12)
4	*Individual-level covariates & area-disadvantage*	0.006 (0.001, 0.014)	0.08	1.08 (1.03, 1.12)
5	*Full model: all individual- & area-level covariates*	0.005 (0.001, 0.012)	0.12	1.07 (1.03, 1.11)

CrI: Credible Interval.

1. Models 2–5 adjusted for all individual-level covariates; Model 4 also adjusted for area disadvantage; Model 5 also adjusted for area remoteness and area disadvantage.

2. The residual area-level variance from the MCMC simulations for multilevel analysis.

3. From Wald χ2 test.

4. Median odds ratio-Refer to text and Appendix 1 for details.

**Table 5:** **Estimated area-level random effects from Bayesian spatial survival models**

		Area random-effects (95% CrI)			
Model	Description ^1^	Spatial (***spat_σ*** ^***2***^_***u***_) ^2^	Unstructured (***spat_σ*** ^***2***^_***v***_) ^3^	Total (***spat_σ*** ^***2***^ ***)*** ^***4***^	Spatial fraction ^5^ (95% CrI)
7	*Null (no covariates) only area random effects: spat_u*_*j*_ & *spat_v*_*j*_	0.018 (0.016, 0.23)	0.006 (0.004, 0.012)	0.024 (0.012, 0.28)	0.70 (0.51, 0.82)
8	*Individual covariates*	0.010 (0.005, 0.018)	0.005 (0.002, 0.011)	0.015 (0.009, 0.024)	0.64 (0.38, 0.86)
9	*Individual covariates & area remoteness*	0.007 (0.003, 0.015)	0.005 (0.002, 0.010)	0.012 (0.006, 0.021)	0.56 (0.26, 0.82)
10	*Individual covariates & area disadvantage*	0.006 (0.003, 0.13)	0.005 (0.002, 0.011)	0.011 (0.007, 0.019)	0.58 (0.29, 0.81)
11	*Full model: all individual & area covariates*	0.006 (0.002, 0.013)	0.005 (0.003, 0.009)	0.011 (0.006, 0.019)	0.55 (0.35, 0.73)
12	*All covariates with no spatial effect (spat_u*_*j*_ *excluded)*	-	0.009 (0.003, 0.013)		
13	*All covariates with no unstructured effect (spat_v*_*j*_ *excluded)*	0.009 (0.003, 0.014)	-		

CrI: Credible Interval.

1. Models 8 to13 adjusted for all individual-level covariates; Model 9 also adjusted for area remoteness; Model 10 also adjusted for area disadvantage, Model 11 also adjusted for area remoteness and area disadvantage. Models 12 and 13 are adjusted for all covariates in Model 11 but exclude the spatial and unstructured random effects respectively.

2. Spatial variance (*spat_σ* ^*2*^_*u*_).

3. Unstructured variance (*spat_σ* ^*2*^_*v*_).

4. Total variance ().

5. Refer to Appendix 2 for details.

The observed patterns for the main effect parameter estimates generated from the two modeling approaches were broadly similar (Table 6), although the CrI of the multilevel estimates were generally equal to or wider than those for the Bayesian spatial model. As expected, within those categories with large numbers of deaths (e.g. Stage IV cancers), there were large differences in the OR and RRs estimates due to the violation of the rare disease assumption when using ORs to estimate RRs.

**Table 6:** **Covariate fixed effects from multilevel discrete-time and Bayesian spatial survival models**

Variable	Multilevel model: OR (95% CrI) ^1^	Spatial model: RR (95%CrI) ^2^
**Area-level variables**		
***Area-Remoteness Index of Australia***		
Major city	**1.00**	**1.00**
Inner regional	0.95 (0.88, 1.02)	0.98 (0.89, 1.07)
Outer regional	1.09 (1.01, 1.18)	1.06 (1.01, 1.19)
Remote	1.15 (1.02, 1.28)	1.09 (1.01, 1.21)
***Relative socioeconomic advantage and disadvantage***		
Most advantaged	**1.00**	**1.00**
Advantaged	1.14 (1.03, 1.23)	1.08 (1.01, 1.17)
Middle	1.18 (1.08, 1.29)	1.15 (1.06, 1.25)
Disadvantaged	1.22 (1.11, 1.34)	1.17 (1.07, 1.28)
Most disadvantaged	1.23 (1.10, 1.36)	1.18 (1.07, 1.32)
**Individual-level variables**	***modeled at unit record***	***modeled as aggregated data***
***Age group***		
20 to 49	0.24 (0.21, 0.27)	0.29 (0.26, 0.32)
50 to 59	0.29 (0.26, 0.32)	0.35 (0.32, 0.38)
60 to 69	0.42 (0.39, 0.46)	0.48 (0.45, 0.52)
70-79	0.68 (0.63, 0.73)	0.73 (0.69, 0.77)
80-85	**1.00**	**1.00**
***Gender***		
Male	**1.00**	**1.00**
Female	1.08 (1.02, 1.14)	1.07 (1.02, 1.13)
***Marital status***		
Married	**1.00**	**1.00**
Never married/single	1.33 (1.21, 1.46)	1.31 (1.20, 1.40)
Widowed	1.11 (1.03, 1.19)	1.09 (1.02, 1.15)
Divorced	1.18 (1.08, 1.29)	1.16 (1.08, 1.25)
Separated	0.94 (0.77, 1.13)	0.95 (0.81, 1.15)
Not stated	1.32 (1.02, 1.68)	1.36 (1.08, 1.69)
***Occupation category***		
Professional	**1.00**	**1.00**
White collar	1.11 (1.02, 1.20)	1.07 (1.01, 1.15)
Blue collar	1.38 (1.29, 1.49)	1.29 (1.22, 1.37)
Not in labor force	0.46 (0.43, 0.50)	0.52 (0.49, 0.56)
Not stated/Inadequately described	0.35 (0.32, 0.39)	0.39 (0.35, 0.42)
***Country of birth***		
Australia	**1.00**	**1.00**
Other English-speaking	0.96 (0.90, 1.02)	0.95 (0.92, 1.00)
Non-English-speaking	0.88 (0.76, 0.97)	0.87 (0.78, 0.98)
***Indigenous status***		
Non Indigenous	**1.00**	**1.00**
Indigenous	1.16 (0.89, 1.49)	1.12 (0.97, 1.38)
Not stated	0.45 (0.39, 0.51)	0.48 (0.42, 0.54)
***Site***		
Proximal (R) colon	1.02 (1.01, 1.08)	1.06 (1.01, 1.11)
Distal (L) colon	1.03 (0.96, 1.10)	1.07 (1.01, 1.13)
Colon NOS	1.04 (1.01, 1.16)	1.08 (1.00, 1.18)
Rectal	**1.00**	**1.00**
***Stage***		
Stage I	**1.00**	**1.00**
Stage II	1.61 (1.47, 1.77)	1.57 (1.44, 1.71)
Stage III	3.17 (2.91, 3.45)	2.85 (2.64, 3.10)
Stage IV	11.41 (10.30, 12.57)	7.88 (7.23, 8.59)
Unknown stage	2.09 (1.86, 2.34)	2.10 (1.91, 2.32)
***Differentiation***		
Well differentiated	**1.00**	**1.00**
Moderately differentiated	1.14 (1.01, 1.29)	1.18 (1.06, 1.32)
Poorly differentiated	1.64 (1.44, 1.87)	1.65 (1.47, 1.85)
Not stated differentiation	1.25 (1.09, 1.43)	1.35 (1.20, 1.52)
***Surgical margins***		
Clear	**1.00**	**1.00**
Positive	1.42 (1.19, 1.66)	1.37 (1.19, 1.57)
Unknown margin	1.84 (1.68, 2.01)	1.73 (1.61, 1.85)

CrI Credible Interval OR Odds Ratios, RR Relative Risk ratios.

1. Estimates derived from best fitting fully adjusted Model 5 as described in text.

2. Estimates derived from best fitting fully adjusted Model 11 as described in text.

## Discussion

To our knowledge this is the first report of a case-study approach to explore the implications of using multilevel discrete-time [7, 8] and Bayesian spatial survival models [4, 5] for the same population-based cohort. These complex models were estimated using MCMC methods to reduce estimation bias for multilevel discrete-time models [7] and produce more reliable small-area estimates for spatial analyses [14].

Through a systematic comparison of the two approaches this study highlights important differences between the multilevel and spatial perspectives in analyzing cancer survival including model specification, underlying concepts, assumptions regarding model-effects and interpretation of area-level random effects in the context of population-based data that typically cover numerous geographical areas and have long term follow-up.

While the fixed estimates from the two approaches cannot be compared directly [23], we found that adjusting for within- or between- area clustering had only a minimal impact on the broad patterns for the fixed estimates. For example, people from remote areas had poorer all-cause survival than those from major cities for both approaches. This is consistent with a recent simulation study that found fixed effects were similar for multilevel and spatial methods [17].

A key feature of the multilevel approach is its ability to relate the estimated geographical variation to the total survival differences between individual patients. A number of additional parameters have also been developed for multilevel logistic regression, such as the MOR, which uses the estimated area-level random effect to quantify the median variability in survival between two randomly selected patients from two different areas with identical individual-level characteristics [10, 44]. However there is a lack of well accepted and robust measures for reporting the magnitude and impact of small-area variation in survival from Bayesian spatial models in a meaningful manner [45]. Tango’s MEET [46] is a global clustering test that has been previously used to formally evaluate the significance of the modelled spatial variation in Bayesian spatial survival results [4, 37], but computational difficulties with the large number of variables in our models precluded this approach here.

An important strength of the Bayesian spatial models adopted for this case study is their ability to account for spatial associations while borrowing information from neighboring areas to enable stable small-area estimates. Using aggregated spatial models potentially also allows greater flexibility in incorporating more years whereas the multilevel model requires curtailing the data. Moreover, the Bayesian spatial model, unlike the multilevel discrete-time model, can be easily modified to conduct relative survival analyses [4, 5], the preferred approach when reporting population-based cancer survival estimates [47].

There are also limitations to both approaches. Multilevel discrete-time survival analysis requires an initial restructuring to the *person-period* format so that standard binary response regression can be carried out [8]. Given the size of our primary dataset, the additional expansion required for analyzing survival outcomes over the entire time period or with shorter time intervals (e.g. days, months) rather than years was not possible under our computing specifications. This is a key limitation of MLwiN, which, as the most widely used software for multilevel modeling, may make this approach computationally infeasible [48]. Parameter estimates from continuous time survival models have however been shown to be comparable to those from multilevel discrete-time survival models [3, 8, 49]. For the Bayesian spatial model, the estimates are based on data aggregated by geographical units; hence making inferences at the individual level are subject to the well-known ecological bias [14, 50]. Both models when run under the computationally intensive MCMC were very time-consuming. An alternative option could be to use the R package INLA (Integrated Nested Laplace Approximation) [51] to generate results instead. This method approximates fully Bayesian inference and generates within seconds or minutes rather than days, but is only available for selected models [52].

Given the differences between the two approaches, the choice of analytic methods will depend on the research questions of interest, data characteristics, and available computational resources. Multilevel models may be more appropriate if a clear hierarchical structure is apparent and the primary objective is to quantify the independent impact of individual and area-level factors on survival differences while accounting for the clustering at the different analytical levels. Spatial analyses may however be preferred if the spatial correlation between areas has a theorized impact on the observed inequalities, or if the goal is to study geographical variation in cancer survival at the small-area level and then create maps of the smoothed relative risk estimates to understand spatial patterns. Such maps can prove useful in identifying areas with lower survival (or elevated relative risk of mortality) relative to all other regions within the overall study area [4, 5] with the potential to guide targeted strategies for improving survival and allocating resources.

The approaches described in the current study are generalizable in terms of wider international settings, geographical units (i.e. not restricted to SLAs) and cancer sites that can be analysed. These models can be fitted to datasets from any population-based or hospital-based cancer registry provided that there is sufficient information to estimate survival and assign cases to a geographical unit. Finally these models can be readily extended to look at geographical inequalities in survival for other diseases and conditions than cancer.

## Conclusions

As spatial models more accurately define the geographical composition with the study cohort by accounting for spatial proximity, perhaps the optimum approach would be to integrate these two approaches by combining the spatial structure and neighboring information with a multilevel survival model that retains the nested structure. Literature on incorporating a spatial perspective into the multilevel setting is comparatively rare [18–21]. Various conceptual and technical challenges have limited the easy implementation of multilevel spatial models in practice including their inherent complexity, computational demands and concerns about the statistical stability and interpretation of model estimates [19–21]. This may be a promising area for further research.

## Appendix 1 Multilevel discrete-time survival analysis

Multilevel discrete-time survival models [8] were adopted to analyse geographical variations in five year all-cause survival for individual patients. As described elsewhere [3], this approach requires an initial expansion of the dataset to allow survival models to be specified as multilevel binary response models.

### Data expansion

We used the death or censoring time, *r*_*ij*_, and an indicator *δ*_*ij*_ which was 0 if death had not occurred and 1 if death had occurred by five-years for each individual *i* in the *j*^*th*^ SLA in the original data, to create for each follow-up interval *t* (years) up to *r*_*ij*_ a binary response *e*_*tij*_ which was coded as:


Hence if an individual died during the third year after diagnosis their discrete responses were (e_1ij_, e_2ij_, e_3ij_) = (0,0,1), while someone who was censored in the third year had response vector (0,0,0). This restructured dataset is often referred to as a *person-period file* [8].

### Priors

The intercept and fixed parameters were assigned diffuse uniform priors (mean 0, variance 1.0). A weakly informative hyperprior of *Gamma* (0.1, 1000) was used for the precision *ml*_*τ*_*u*_ (inverse variance) on the area-level random effect *ml*_*u*_*j.*_ These are the default prior distributions in MLwiN [22]. Given the large number of area level units (478 SLAs) inferences are unlikely to be sensitive to the choice of prior distributions for the area-level variance [53].

### Median odds ratios

The median odds ratio (MOR) was calculated as described previously [10]:


where Ζ_0.75_ is the 75^th^ percentile of the normal distribution and *ml*_*σ*^*2*^_*u*_ is the estimated area-level variance from the MCMC simulations. A 95% credible interval for the MOR was generated from the posterior distribution of the variance [43].

## Appendix 2 Bayesian spatial survival analysis

### Priors

An exchangeable normal prior *spat_v*_*j*_ ~ N(0,*spat_σ*^*2*^_*v*_) was specified for the non-spatial random effect where *spat*_*σ*^*2*^_*v*_ is the variance. The spatial dependence (*spat*_*u*_*j*_) across SLAs was estimated using an intrinsic conditional autoregressive (CAR) prior [31] defined as:


where *ω*_*jk*_ =1 if j, k are adjacent SLAs and 0 otherwise and *spat_σ*^*2*^_*u*_ is the variance for the spatial effect. Neighbors were defined using an adjacency matrix as described previously [37]. Diffuse normal priors were used for the intercept and fixed effects and weakly informative Gamma hyperpriors for the precision parameters *spat*_*τ*_*u*_ and *spat_τ*_*v*_.

Sensitivity analyses were conducted by specifying three different Gamma (Γ) distributions for *spat*_*τ*_*u*_ and *spat_τ*_*v*_ and two uniform (Unif) priors for the standard deviation (*spat*_*σ*_*u*_, *spat*_ *σ*_*v*_) [4]:

1. *spat* _ *τ*_*u*_ ~ Γ(0.1, 100), *spat* _ *τ*_*v*_ ~ Γ(0.1, 100)
2. *spat* _ *τ*_*u*_ ~ Γ(0.5, 1000), *spat* _ *τ*_*v*_ ~ Γ(0.5, 1000)
3. *spat* _ *τ*_*u*_ ~ Γ(0.1, 10), *spat* _ *τ*_*v*_ ~ Γ(0.001, 1000)
4. *spat* _ *σ*_*u*_ ~ Unif(0, 10), *spat* _ *σ*_*v*_ ~ Unif(0, 10)
5. *spat* _ *σ*_*u*_ ~ Unif(0, 1000), *spat* _ *σ*_*v*_ ~ Unif(0, 1000)

Priors 1 to 2 had means and variances on the precisions of (10, 1000); (500, 500000); and for Prior 3, *spat*_*τ*_*u*_ had (1, 10), while *spat_τ*_*v*_ had (1, 1000), respectively. Priors 4 and 5 had means and variances on the standard deviations of (5, 8.3) and (500, 83333.3).

Models were compared in in terms of DIC statistics [40], cumulative distribution plots of deviance [54], summary measures of the posterior distribution of monitored parameters and convergence diagnostics.

### Spatial fraction

If *spat_σ*^*2*^_*u(m)*_ is the marginal variance for the spatial effect and *spat_σ*^*2*^_*v*_ is the variance for the non-spatial effect then the spatial fraction (Ψ) [5] is:


## Electronic supplementary material

Additional file 1: **Example of sensitivity analysis for Bayesian spatial survival models.** Kernel density plots for estimated relative risks (RR) of all-cause death by area-level remoteness: A: major cities; B: inner cities and C: remote from Bayesian spatial survival models with Gamma priors specified for the precision (inverse of variance). of 1: τ_u_ ~ Γ(0.1, 10), τ_v_ ~ Γ(0.1, 10); 2: τ_u_ ~ Γ(0.5, 1000), *τ*_*v*_ ~ *Γ*(0.5, 1000); 3 : τ_u_ ~ Γ(0.1, 10) τ_v_ ~ Γ(0.001, 1000); or Uniform (Unif) priors on the standard deviation of : 4 : σ_u_ ~ Uniform(0, 10), *σ*_*v*_ ~ Unif(0, 10) or 5 : *σ*_*u*_ ~ Unif(0, 1000), *σ*_*v*_ ~ Unif(0, 1000). (PDF 88 KB)

Additional file 2: **Model comparisons for multilevel discrete-time survival models.** Table S1 shows Bayesian deviance information criterion (DIC) values for different models with smaller values (difference of at least 7 units) indicating better model-fit. Models with difference of 3–5 units can be weakly distinguished. The pD values represents the effective number of parameters. (XLSX 12 KB)

Additional file 3: **Model comparisons for the Bayesian spatial survival models.** Table S2 shows Bayesian deviance information criterion (DIC) values and pD values, which due to borrowing of strength from adjacent areas is often less than the total number of model parameters. Larger values indicate estimates have undergone less smoothing. (XLSX 12 KB)

Additional file 4: **Median smoothed relative risk (RR) and credible intervals by statistical local areas (SLA).** The median smoothed relative risk (RR) estimates from final Bayesian spatial model for all-cause survival by statistical local areas (SLAs) in Queensland. The black line is the RR, grey lines are the 95% credible intervals (CrI) and the red horizontal line indicates the Queensland average. (PDF 52 KB)

## Abbreviations

CRC: Colorectal cancer
CI: Confidence interval
CrI: Credible interval
CAR: Conditional autoregressive
DIC: Deviance information criterion
MCMC: Markov chain Monte Carlo
MOR: Median odds ratio
OR: Odds ratio
RR: Relative risk
SLA: Statistical Local Area.
