# Supplementary figures and images for: Comparing multilevel and Bayesian spatial random effects survival models to assess geographical inequalities in colorectal cancer survival: a case study
Source: Int J Health Geogr. 2014 Oct 4;13:36. doi: 10.1186/1476-072X-13-36 (PMC4197252; doi:10.1186/1476-072X-13-36)

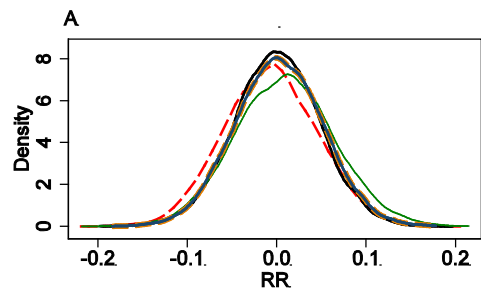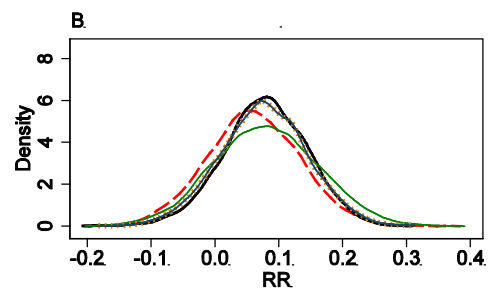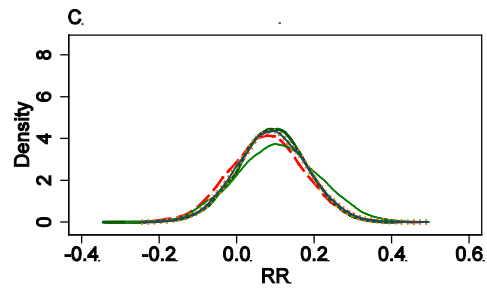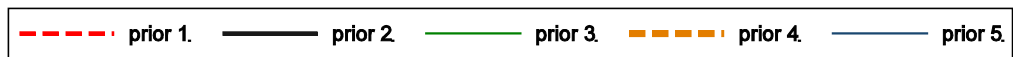

Supplement: Supplementary file 1 — Additional file 1: Example of sensitivity analysis for Bayesian spatial survival models. Kernel density plots for estimated relative risks (RR) of all-cause death by area-level remoteness: A: major cities; B: inner cities and C: remote from Bayesian spatial survival models with Gamma priors specified for the precision (inverse of variance). of 1: τu ~ Γ(0.1, 10), τv ~ Γ(0.1, 10); 2: τu ~ Γ(0.5, 1000), τ v ~ Γ(0.5, 1000); 3 : τu ~ Γ(0.1, 10) τv ~ Γ(0.001, 1000); or Uniform (Unif) priors on the standard deviation of : 4 : σu ~ Uniform(0, 10), σ v ~ Unif(0, 10) or 5 : σ u ~ Unif(0, 1000), σ v ~ Unif(0, 1000). (PDF 88 KB) [file 12942_2014_604_MOESM1_ESM.pdf]

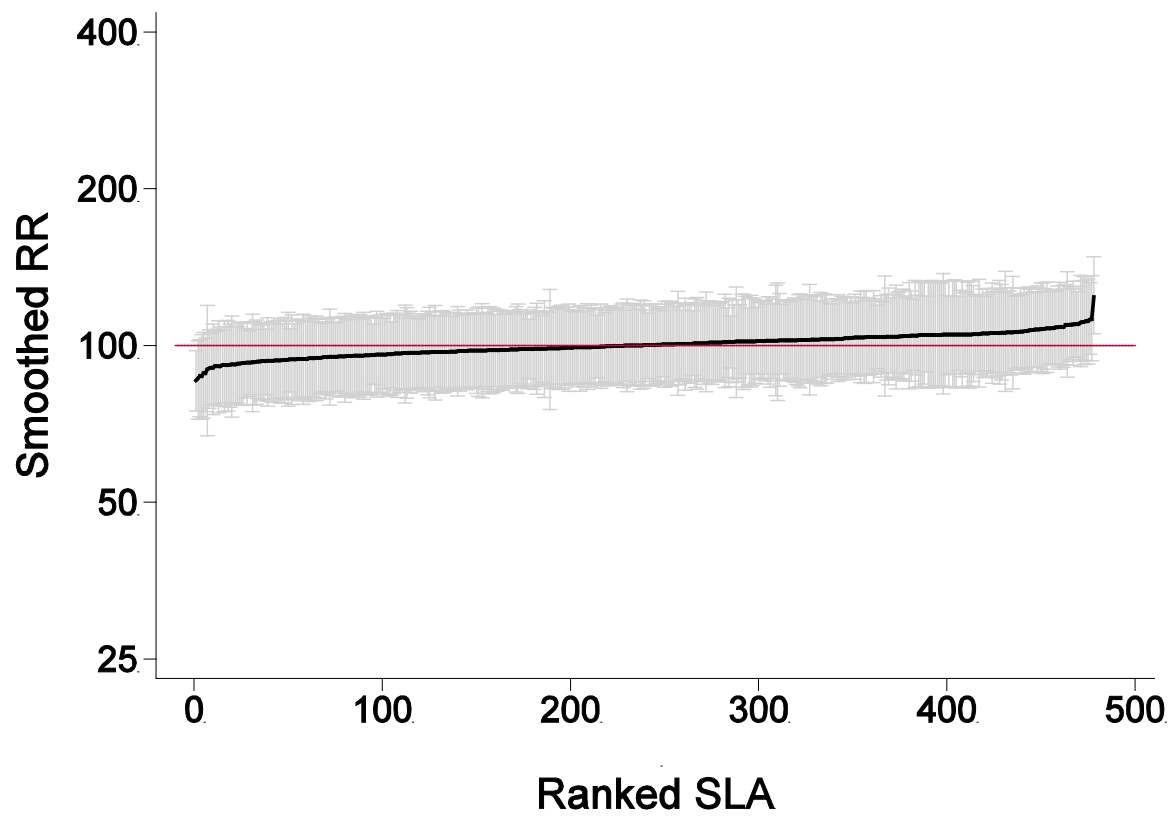

Supplement: Supplementary file 4 — Additional file 4: Median smoothed relative risk (RR) and credible intervals by statistical local areas (SLA). The median smoothed relative risk (RR) estimates from final Bayesian spatial model for all-cause survival by statistical local areas (SLAs) in Queensland. The black line is the RR, grey lines are the 95% credible intervals (CrI) and the red horizontal line indicates the Queensland average. (PDF 52 KB) [file 12942_2014_604_MOESM4_ESM.pdf]
